# Anatase TiO_2_ deposited at low temperature by pulsing an electron cyclotron wave resonance plasma source

**DOI:** 10.1038/s41598-020-78956-1

**Published:** 2020-12-15

**Authors:** B. Dey, S. Bulou, T. Gaulain, W. Ravisy, M. Richard-Plouet, A. Goullet, A. Granier, P. Choquet

**Affiliations:** 1 Materials Research and Technology Department, Luxembourg Institute of Science and Technology, 5 Avenue des Hauts-Fourneaux, 4362 Esch-sur-Alzette, Luxembourg; 2 Institut Des Matériaux Jean Rouxel (IMN), Université de Nantes, CNRS, 2 rue de la Houssinière, BP 32229 44322 Nantes, France

**Keywords:** Materials for energy and catalysis, Photocatalysis, Materials science

## Abstract

Photocatalytic surfaces have the potentiality to respond to many of nowadays societal concerns such as clean H_2_ generation, CO_2_ conversion, organic pollutant removal or virus inactivation. Despite its numerous superior properties, the wide development of TiO_2_ photocatalytic surfaces suffers from important drawbacks. Hence, the high temperature usually required (> 450 °C) for the synthesis of anatase TiO_2_ is still a challenge to outreach. In this article, we report the development and optimisation of an ECWR-PECVD process enabling the deposition of anatase TiO_2_ thin films at low substrate temperature. Scanning of experimental parameters such as RF power and deposition time was achieved in order to maximise photocatalytic activity. The careful selection of the deposition parameters (RF power, deposition time and plasma gas composition) enabled the synthesis of coatings exhibiting photocatalytic activity comparable to industrial references such as P25 Degussa and Pilkington Activ at a substrate temperature below 200 °C. In addition, to further decrease the substrate temperature, the interest of pulsing the plasma RF source was investigated. Using a duty cycle of 50%, it is thus possible to synthesise photocatalytic anatase TiO_2_ thin films at a substrate temperature below 115 °C with a deposition rate around 10 nm/min.

## Introduction

Mankind is currently going through a lot of crucial issues. To cite some, the access of decontaminated air and water is already a critical point in overpopulated countries. The green and efficient generation of H_2_, tomorrow’s fuel, is becoming urgent. The demand in antimicrobial surfaces in hospitals to dam up the spread of diseases is growing^1^. Many studies have already led to the development of materials fulfilling these properties requirements. Among all of them, TiO_2_ stands out from the crowd. As main reasons for this, we can highlight its low price, high availability, chemical stability and non-toxicity for both environment and human beings. Hence, the past century has drawn a lot of attention into this semiconductor that became one of the preferred photocatalyst^2^.

A great variety of deposition techniques can been used to synthesize the most photocatalytic polymorph of TiO_2_, the so-called anatase^3–7^. Depending on the process used, characteristics of the obtained TiO_2_ layers such as grain size and shape, face orientation, porosity, roughness can vary a lot. Main TiO_2_ coatings deposition processes can be divided in two categories namely the wet and dry processes. The former comprises hydrothermal, electrodeposition, anodization, spin-coating, dip-coating and sol–gel methods^8^. Although they represent a cost effective way to synthesise titania layers, wet processes either include a high temperature and/or long post process treatment to get a well crystallized material^9^. Dry vacuum processes such as Physical Vapour Deposition (PVD) or Chemical Vapour Deposition (CVD) also require a step of heating above 200 °C, either during the deposition process or post-deposition (calcination or annealing). This makes them incompatible with the deposition on thermally labile substrates, namely stiff and flexible polymers. For these reasons, up to now, most fundamental and applied studies on TiO_2_ thin film deposition synthesis concerned deposition on glass, which allows deposition temperatures up to 400 °C.

The recent development of flexible electronics and solar cells as well as antibacterial films has created a new demand for materials with transparent conducting or photocatalytic properties which can be deposited at a temperature compatible with most of flexible polymers, typically below 80 °C. Wet processes have been reported to efficiently lead to low temperature photocatalytic thin films synthesis. Hence, Xu et al*.* achieved the deposition of an anatase/rutile mixture by the mean of wet chemistry with an overall process temperature not exceeding 80 °C^10^. Nevertheless, the reaction pathway for such coating involved many steps and long post-treatments. Among them, one can cite a TiO_2_ seeding by sol–gel, then an immersion in Ti-H_2_O_2_ for 72 h at 80 °C. Finally, the crystallization of TiO_2_ was driven by an immersion of the coated PET samples in a 0.05 M H_2_SO_4_ solution at 80 °C for 120 h. Thus, it exists a strong interest in the development of one-step and dry process that can efficiently lead to the rapid deposition of crystalline anatase thin films at low substrate temperature, compatible with thermally labile substrate. This induced the development of new processes to reach highly photocatalytic crystalline anatase TiO_2_ at temperatures far under its thermodynamic crystallisation temperature (250 °C). Only few works focusing on this goal have been published but up to now, the proposed approaches still require long multistep processes^11,12^*.* Thus, mass production of highly photocatalytic thin films deposited at low substrate temperature is still one of nowadays burning issues.

Plasma assisted CVD methods have shown their ability for the synthesis of crystalline inorganic thin films at lower substrate temperature than the one allowed with CVD methods. Indeed, the reactive species generated by the plasma such as energetic electrons, ions, radicals or excited neutrals, can bring energy to the on-growing thin film through different mechanisms other than pure thermal energy. Hence, low pressure PECVD is particularly suited for the synthesis of crystalline functional oxides.

In particular, Electron Cyclotron Wave Resonance plasma sources to assist a CVD process have shown excellent results for the growth of TiO_2_ anatase thin films. Indeed, highly photocatalytic thin films have been obtained at substrate temperature as low as 200°C^13^. Besides, it has recently been shown that pulsing the plasma power can be a convenient way to limit the substrate heating^14–16^, especially for crystalline TiO_2_ thin film growth^17^*.* By carefully setting up the plasma pulse parameters, Li et al*.* obtained anatase containing TiO_2_ layers with good photocatalytic activity while keeping substrate temperature below 120 °C. However, in this Inductively Coupled Plasma (ICP) Radio Frequency (RF) PECVD process, it is worth mentioning that the thickness growth rate caps around 1 nm/min which is not suitable for mass production. In the following work, it is expected that the growth rate of crystalline films could be drastically increased thanks to the high plasma density provided by ECWR plasma source.

In this article, we investigated the possibility of producing, in one-step, crystalline anatase TiO_2_ layers with highly efficient photocatalytic yields, below 120 °C. As a safe, stable and inexpensive precursor, the metal–organic titanium (IV) isopropoxide (TTIP) was chosen as titanium precursor. In addition to the optimisation of the main experimental parameters (power, deposition time and gas composition), we especially focus on the pulsing of the Electron Cyclotron Wave Resonance (ECWR) plasma source in order to perform the deposition at low substrate temperature.

## Results and discussion

**Figure 1:**
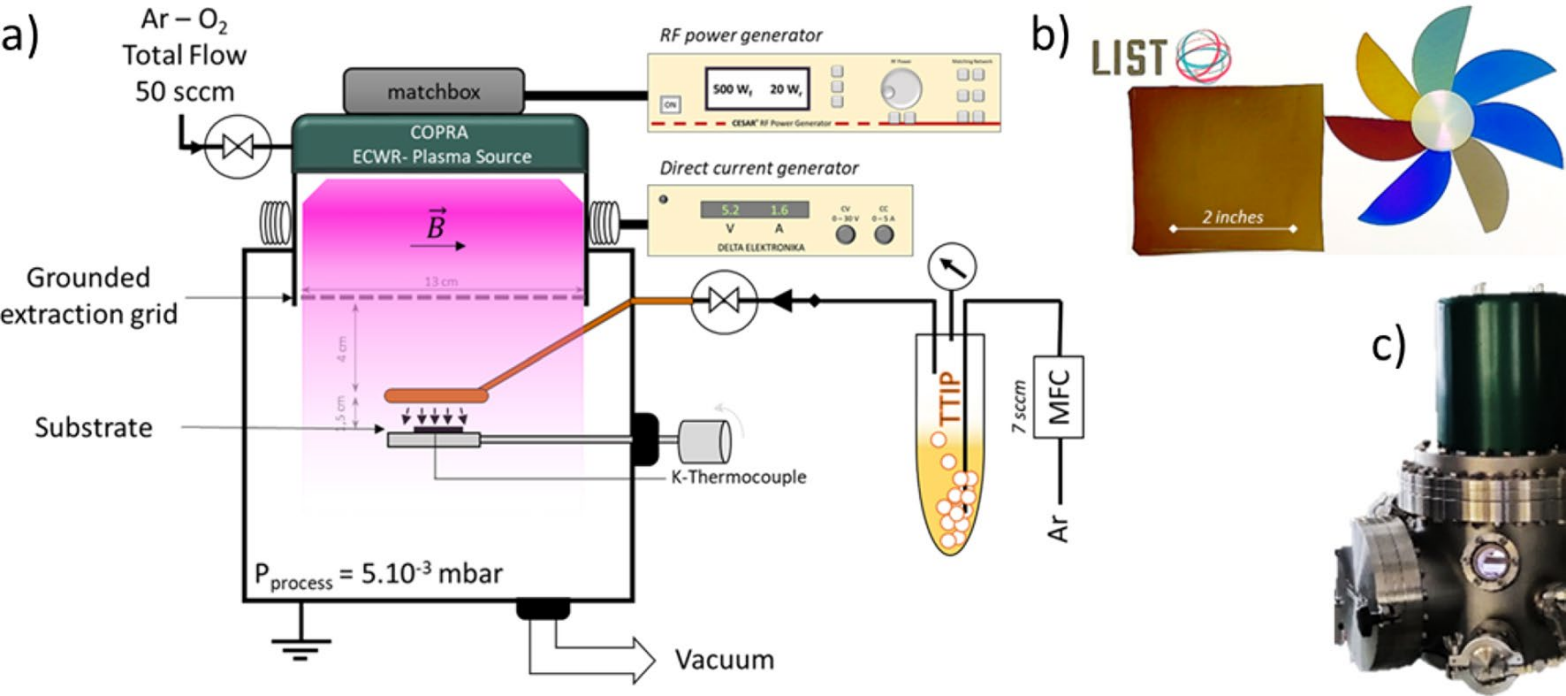
Scheme of the experimental setup of ECWR Plasma process (**a**), TiO_2_ thin films obtained with Copra DN 200 CF plasma source for different thickness (**b**) and picture of the source igniting an Ar–O_2_ plasma at 500 W (**c**).

In the following study, several parameters linked to the chemistry and the plasma process were discussed. For the ease of understanding, we decided to focus only on the influence of specific deposition parameters. The constant parameters linked to the deposition set-up for the results presented in this article are summarized in methods section. As depicted on Fig. 1a, the injection ring was placed really near the substrate (1.5 cm) in order to foster surface reactions and limit gas phase reactions. The plasma source generates a quasi-neutral plasma beam allegedly homogeneous over 13 cm diameter.

Therefore, homogeneous samples up to two inches can be coated (Fig. 1b). Moreover, tuning some plasma parameters enabled the deposition of crystalline TiO_2_ on polyethylene substrates with a good adhesion (Fig. 1b) which will be further discussed in the last part of this manuscript. Before going through the different parameters study, one has to notice that XPS analysis pointed out the similar atomic composition of all the synthesised layers presented in this study. Whatever the experimental conditions, the bulk atomic ratio O/Ti is about 1.8. Considering the preferential sputtering of O over Ti under Ar^+^ etching beam this ratio is typical of stoichiometric TiO_2_. In addition, the decomposition of Ti 2p_1/2_ and Ti 2p_3/2_ peaks exhibits a single contribution at 458.9 and 464.7 eV respectively. These characteristic values reveal the sole presence of Ti^4+^ oxidation state in the synthesised films^18,19^. Finally, all the bulk analysis pointed out C content below 4% irrespective of the plasma conditions.

### Influence of the RF power

In this first section, the effect of the continuous RF plasma power on the morphology, composition and crystallinity of the titanium dioxide films and on the substrate temperature increase were investigated. The deposition time was kept at 2 h for this power study (100, 300, 500 W). An Ar flow of 37.5 sccm and an O_2_ flow of 12.5 sccm were used so that the gas flow ratio in the Ar–O_2_ mixture (plasma source), [O_2_]/([O_2_] + [Ar]) was set to 25%. For all these three deposition conditions, the thickness growth rate of the layers seemed independent of the plasma power and cap around 11 ± 1 nm/min and this for a constant TTiP flowrate calculated around 0.16 sccm.

**Figure 2:**
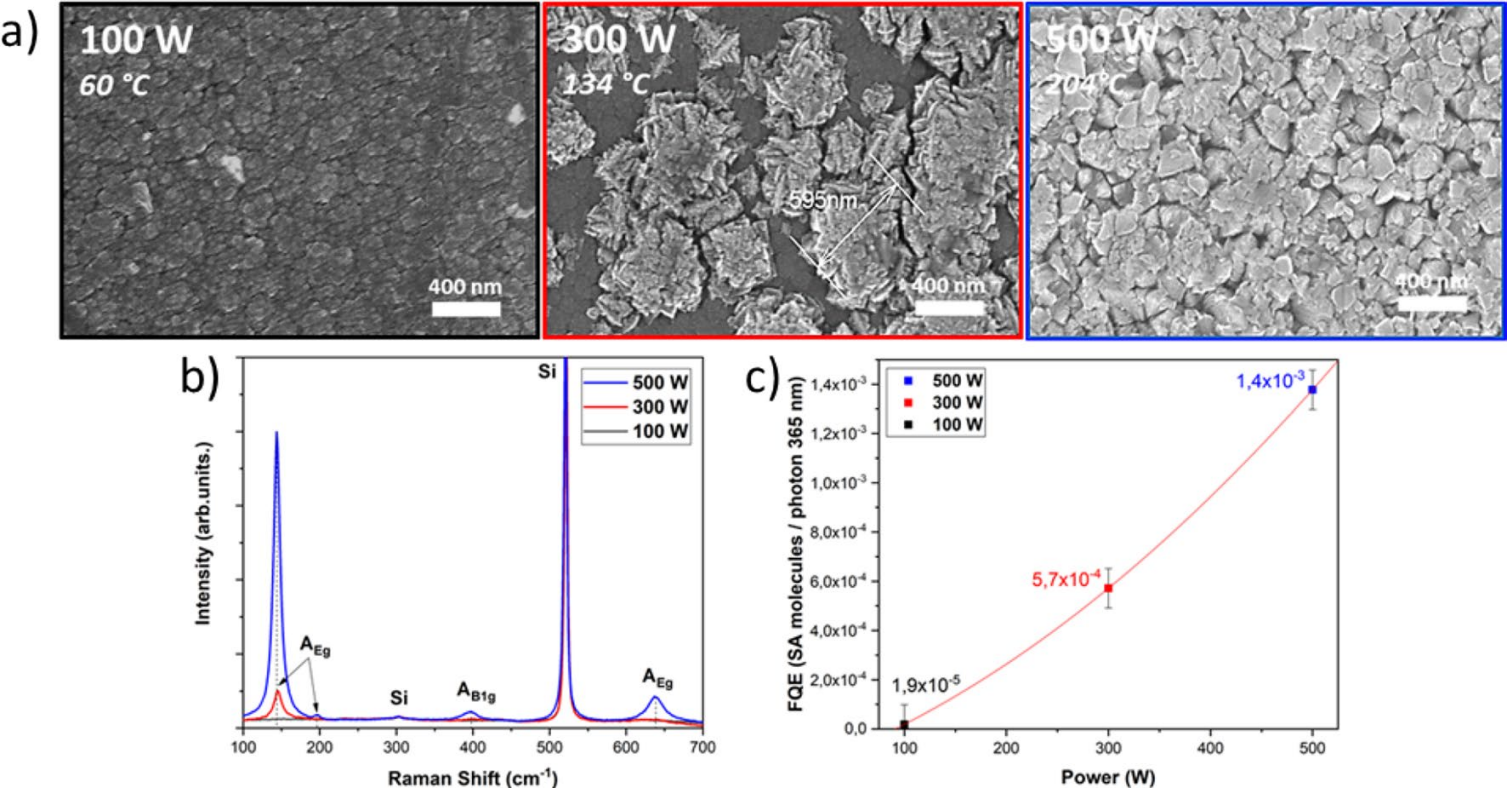
SEM topviews with associated T_sub max_ (**a**), Raman scattering spectra (**b**) and Photocatalytic activity (**c**) of TiO_2_ deposited at different RF power (2 h CW mode deposition).

From these results, we could infer that in these conditions, where the TTiP precursor can be considered as highly diluted in the Ar–O_2_ mixture, the plasma density is high enough, even at 100 W, to fully dissociate TTiP, so that the growth rate did not depend on the plasma power. Besides, it is observed that the final temperature of the substrate T_sub max_ is directly linked to the RF power and a linear relation can be established between these two parameters. The investigation of the coatings surface by SEM, exhibited different morphologies for the titanium dioxide films synthesised at different P_RF_ (Fig. 2).

At P_RF_ = 100 W, a homogeneous surface morphology composed of one single structure could be observed by SEM and the final deposition temperature reaches 60 °C. Taking into account this very low deposition substrate temperature of the titanium dioxide layer and results already reported in the literature, it is possible to put forward that these layers have developed a fibrous structure along the thickness^20^. Based on measurements carried out on the SEM pictures, the diameter of the top of these fibres was estimated around 50 nm. Regarding the structure analysed by Raman scattering (Fig. 2) no specific peak was detected and thus, suggesting an amorphous structure. Nevertheless, it is also possible that the crystallites are too small to be detected by Raman^21^.

As the RF power was increased from 100 to 300 W, an increase in the maximum substrate temperature was recorded (T_sub max_ = 134 °C). In addition, a second structure on the TiO_2_ top surface emerged out from the amorphous fibrous one. This structure presented wide spread “flower-like” microstructures exhibiting sharp edges, partially covering the amorphous phase. These flower-like structures had various sizes, between 300 and 500 nm. The associated Raman analysis shows the appearance of peaks associated to the anatase phase, thus suggesting this flower like structure to be partially crystallised. This could result from a crystal germination mechanism which possibly appeared because of the plasma power increase or/and the higher deposition temperature.

As the plasma power was further increased, an evolution of the surface coating morphology was observed and the substrate temperature increased. At P_RF_ = 500 W, the flower-like structure covered the whole film surface made out of highly faceted grain starting to merge to each other and the T_sub max_ was slightly up to 200 °C which was consistent with our previous published results^13^. Moreover, the intensity of Raman peaks associated to the anatase phase tremendously increased.

Similarly, great differences on the TiO_2_ coatings photocatalytic properties were noticed depending on P_RF_. Thus, the photocatalytic activity of the layers deposited at 100, 300 and 500 W were established. For these three plasma powers, the calculated Formal Quantum Efficiency (FQE) (Eq. 1) values are equal to 1.9 × 10^–5^, 57 × 10^–5^ and 140 × 10^–5^ SA molecules/photon 365 nm, respectively (Fig. 2). Compared to what Noimark et al*.* obtained (FQE_TiO2_ = 7.2 × 10^–5^ and FQE_TiO2/Au_ = 15 × 10^–5^)^22^ or to industrial references in the field such as FQE_P25 Degussa_ = 15.3 × 10^–5^ and FQE_Aktiv_ = 0.7 × 10^–5^*,* these efficiencies stand at the state of the art^23^. The photocatalytic efficiency of these three layers deposited at 100, 300 and 500 W seemed independent of their thickness (1297, 1322 and 1340 nm respectively) but rather linked to the density and or the quality (defects, grain size, main facet) of anatase containing crystals directly in contact of Stearic Acid molecules. The best FQE value was obtained at P_RF_ = 500 W and this statement was well correlated to the huge anatase Raman peaks.

**Figure 3:**
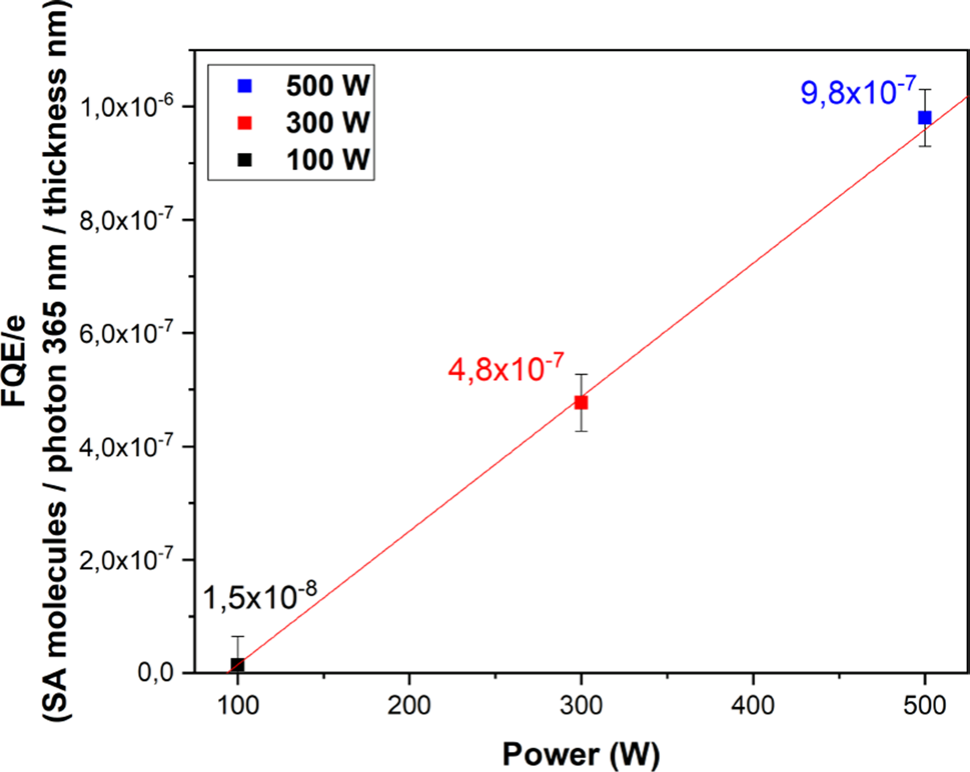
: Normalized photocatalytic efficiency (SA molecules/photon 365 nm/coating thickness) as a function of RF Power (W).

One could hypothesize here that the excellent FQE measured from our as-grown TiO_2_ layers could partly be explained by their quite important thickness (about 1300 + /− 50 nm whatever the power). Thus, a FQE normalized to the film thickness (i.e. FQE/e) can be calculated which could be a simple way to compare the normalized photocatalytic efficiency (SA molecules.photon 365 nm^−1^ coating thickness nm^−1^) of the films grown irrespective of thickness effect (Fig. 3).

Thus by normalizing the Formal Quantum Efficiency by the film thickness, one can more easily compared these plasma deposited TiO_2_ to the above-mentioned reference with FQE/e_Aktiv_ = 4.7 × 10^−7^ nm^−1^.

### Influence of film thickness

**Figure 4:**
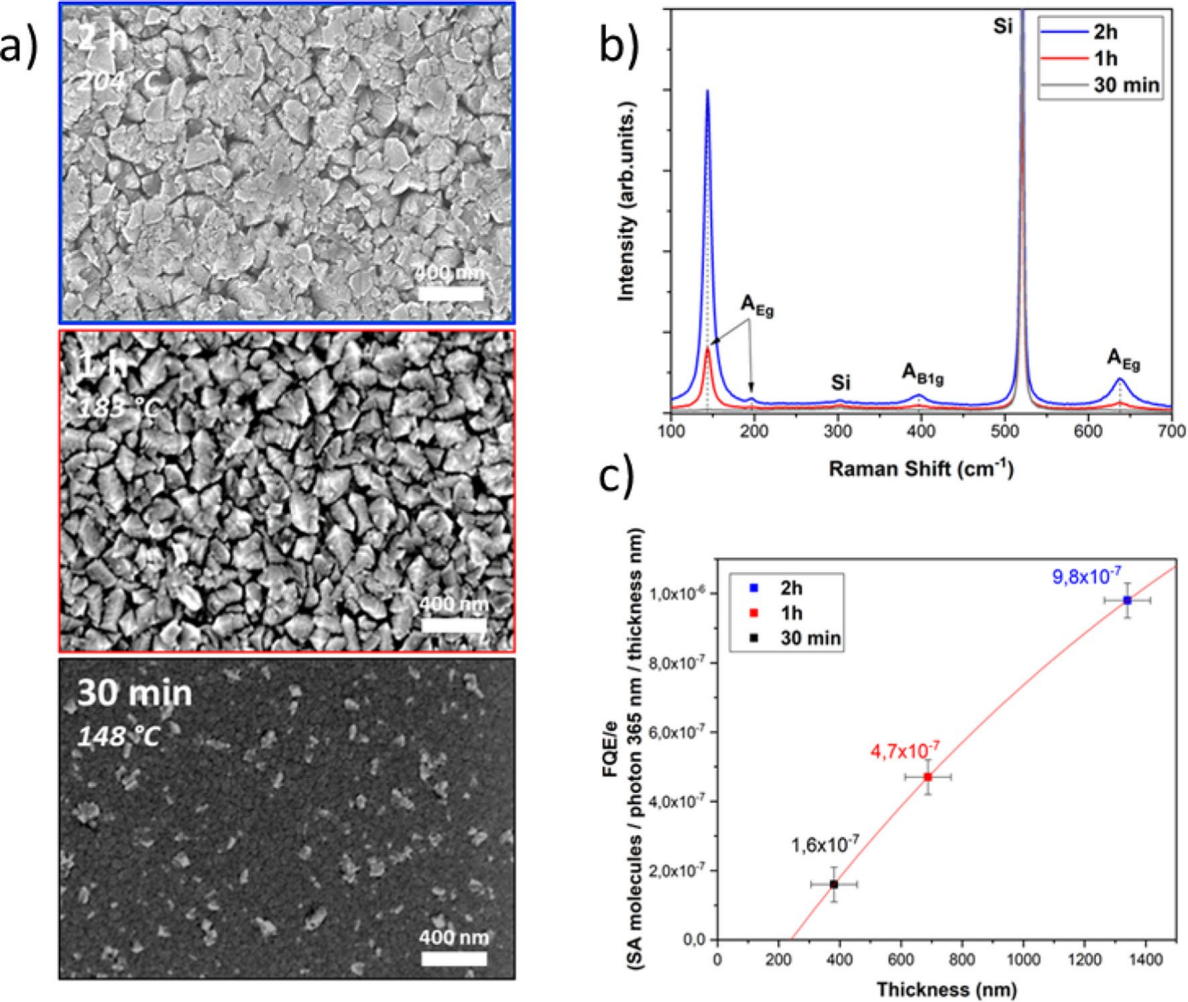
: SEM topviews with associated T_sub max_ (**a**), Raman scattering spectra (**b**) and Photocatalyic activity normalized to the thickness (**c**) of TiO_2_ thin films deposited for different time (CW mode at 500 W).

In order to enlighten the influence of the layer thickness on the photocatalytic activity, the RF plasma power was fixed at 500 W as it demonstrated the best ability for the synthesis of anatase, and the deposition time was varied to get different film thicknesses. Three deposition times were then considered: 30 min, 1 h and 2 h, which lead to film thicknesses of 381, 668 and 1340 nm, respectively. The corresponding maximal substrate temperatures (temperature reached at the end of the deposition process) of 148, 183 and 204 °C respectively were measured. Indeed, as it has been already reported by several authors^24–26^, plasma induced an increase of substrate temperature with time until they reach a certain threshold. For these plasma conditions, the threshold was reached after three hours with an equilibrium temperature of 230 °C. By fitting the data with a linear function, the slope gave a thickness growth rate of 10.7 ± 0.4 nm/min which was in agreement with the value given in the previous section. According to the constant growth rate whatever the deposition time, one can conclude that the substrate temperature does not influence the TiO_2_ deposition speed. The top view SEM investigations of the films surface provided information about the growth mechanism of the TiO_2_ layer as a function of time (Fig. 4). For 30 min deposition time, whereas no crystalline TiO_2_ Raman scattering peaks are observed in Fig. 4, the surface coating exhibits the two morphologies described above. The dark phase associated to the fibrous morphology seems to be similar to the one observed for deposition obtained at P_RF_ = 100 W. Consequently, if the second morphology with sharp edges appearing in bright on SEM micrographs is associated to anatase crystallites, their size or amount was probably too small and could not be detected in the Raman spectra^27^.

Increasing the deposition time up to 1 h led to surface film composed of sharper edges grains with size about 140–160 nm. The Raman spectra on this film revealed several characteristic peaks of the anatase phase.

Whereas it is commonly admitted that only the topmost TiO_2_ layer (i.e. the layer that is directly in contact with the environment) takes part to the photocatalytic reactions, one can notice that an increase of the film thickness (Fig. 4) has led to an enhanced photocatalytic efficiency. However, for these experiments, even for the shortest time, the thickness of the coating reached ≈ 400 nm. According to the literature, it is usually found that the photocatalysis reaches an optimum efficiency for thin films around 500–600 nm^28^. Above this film thickness, the photocatalytic property even starts to decrease^29^. As reported in Fig. 4 on the Raman spectra and considering in a first approximation that the Raman intensity is proportional to the amount of anatase, it can be noted that this allotropic variety of interest for Photo Catalytic Activity (PCA), is raising with the thickness, thus providing more active sites for the photogeneration of electron^−^/hole^+^ pairs required for PCA^30^*.*

As presented above, the plasma power injected into the ECWR plasma source and the thickness of the titanium dioxide film have a strong impact on the surface morphology layer, the degree of crystallinity of the TiO_2_ and consequently, on the photocatalytic properties of the coated substrates. With this particular plasma source, at a pressure set constant, ionic current density is proportional to the power coupled to the plasma. Thus, to carry out the deposition of anatase thin films, it seems necessary to run the plasma at high power to produce a sufficiently high ionic current^31^*.*

At this stage, it is interesting to report that the highest RF plasma power of 500 W and a deposition time above one hour were the best features to produce high yield photocatalytic films. However, if the substrate is not in contact with a highly efficient cooling substrate holder during the deposition, these two process conditions led to an increase of the substrate temperature above 180 °C. This constitutes a major drawback for the deposition on complex substrate shapes or roll-to-roll in line coating processes. Thus, in order to maintain a good photocatalytic property while reducing substrate temperature, one option is to switch from a continuous to a pulsed mode deposition.

### Influence of pulsed mode

In the following, the plasma power was kept at a constant P_RF_ = 500 W during T_on_. This power was chosen since in the previous section, anatase TiO_2_ layer with interesting deposition rate and high photocatalytic property FQE/e_1h 500 W_ = 4.7 × 10^–7^ nm^−1^ were obtained. The aim was to assess the possibility of decreasing the substrate temperature while keeping a high photocatalytic activity by pulsing the plasma source. In the following, a pulse frequency of 10 kHz was used (T_ON_ + T_Off_ = 0.1 ms) and different duty cycles (DC = T_ON_/(T_ON_ + T_Off_)) were investigated (DC = 25, 50,75 and 100%). As expected, tuning down the duty cycle from 100% (CW) to 25% helped to reduce the substrate temperature from 183 to 76 °C. For deposition lasting 1 h, TiO_2_ thin films thickness were measured at 880, 860, 680 and 668 nm for duty cycles (DC) of 25, 50, 75 and 100% respectively.

**Figure 5:**
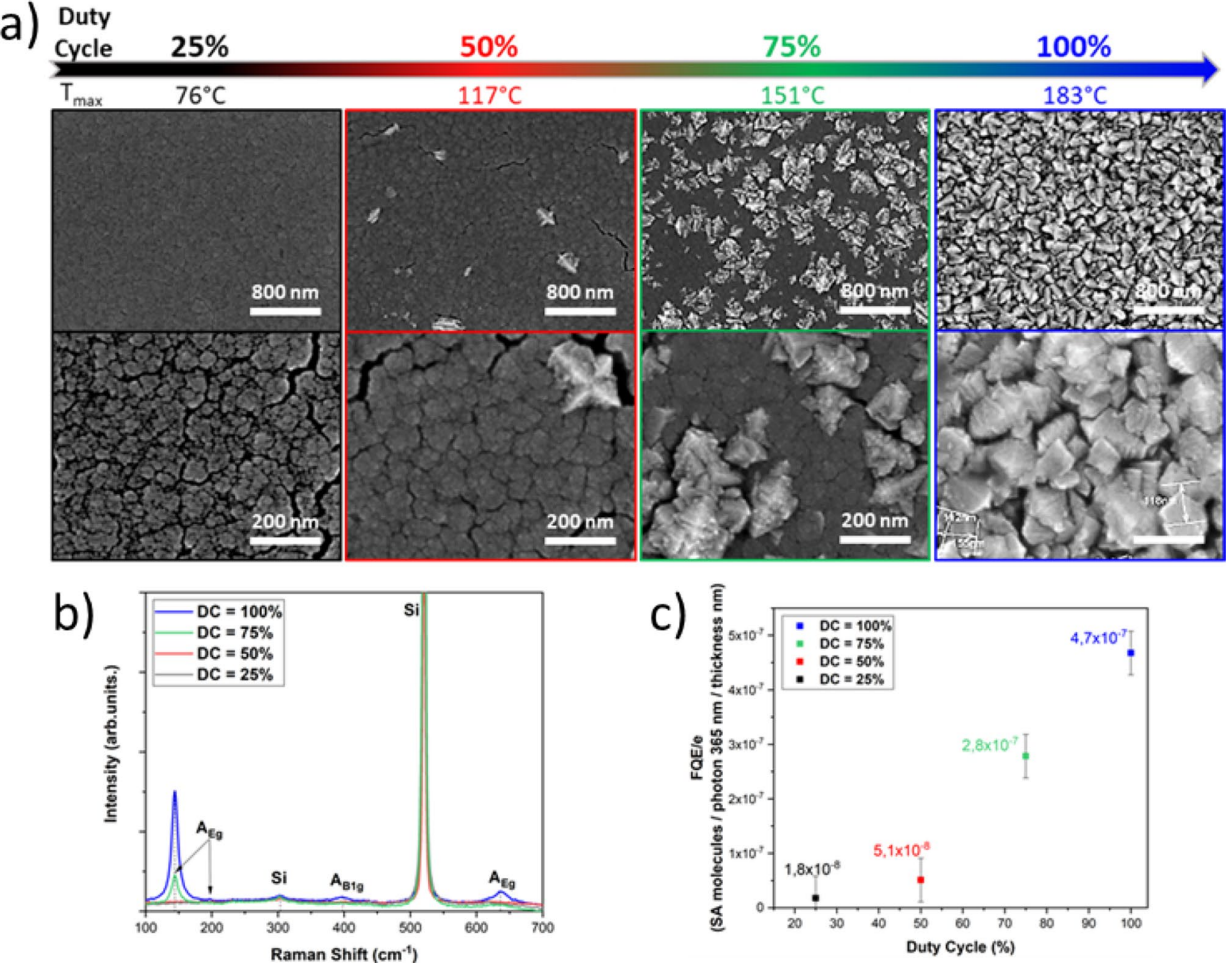
SEM topviews with associated T_sub max_ (**a**) Raman scattering spectra (**b**) and Photocatalytic activity normalized to the thickness (**c**) of TiO_2_ films deposited at different Duty Cycles (Power 500 W (T_on_), 1 h deposition, f = 10 kHz).

A drastic decrease of the Raman peaks associated to anatase is observed with decreasing the DC (Fig. 5).

From SEM surface observations (Fig. 5), important modifications of film surface morphology could be noticed. For the lowest duty cycle investigated (DC = 25%), a morphology similar to the one obtained at continuous P_RF_ = 100 W was observed. Increasing the duty cycle up to 50%, led to two different structures containing surfaces. However, Raman scattering measurements did not evidence significant anatase peaks. The duty cycle had to be tuned up to 75% so the anatase could be spotted out by Raman analysis. At that point the flower like structure was not covering the entire surface but the substrate temperature was already exceeding 150 °C.

In addition, evaluation of the photocatalytic property spotted out moderate PCA, consistent with the SEM observations and Raman analysis suggesting that no or low density of anatase crystals arising at the film surface were noticeable on the films obtained in pulsed mode (Fig. 5). Indeed, when DC ≤ 50% was applied, TiO_2_ layer FQE/e was one order of magnitude lower than the one obtained for DC = 100% (continuous mode). Hence, the thickness normalised yield efficiency (FQE/e) of these layers (DC < 50%) (Fig. 5), was estimated to 2 × 10^–8^ and 5 × 10^–8^ nm^−1^, for DC = 25% and DC = 50% respectively.

For a pulse frequency of 10 kHz, it can be noticed that the associated deposition rates in pulsed mode with the two different duty cycles were very close and even slightly higher than the rate determined in continuous plasma condition. This statement is in good correlation with Charles et al*.*^32^ who performed SiO_2_ deposition in pulsed plasma helicon diffusion out of SiH_4_/O_2_ mixture. They showed that when the pulse frequency was f > 10^2^ Hz, the growth rate was the same as for the continuous process. The conclusion was that deposition mechanism was continuing even during plasma T_Off_. However in the present study, it appeared that the growth rate of the TiO_2_ films obtained in pulse mode was slightly higher than the one under continuous discharge. This phenomena has been shown by other research groups in the case of pulsed PECVD deposition of silicon based films^33^. They ascribed this behaviour to a better use of the precursor during the deposition, by hampering homogeneous gas phase reactions. Thus, by pulsing the plasma these authors reduced such homogeneous reaction. However, this hypothesis seems very unlikely in our deposition conditions since no dust could be found. In addition, as depicted in the first section, highly diluted plasma condition with a flow rate of TTiP around 0.16 sccm so we assumed that even for 100 W the plasma density was high enough to fully dissociate it. Besides, Charles et al*.* demonstrated that adjusting the length of the discharge and the post-discharge had an influence on film internal stress^34^. Thus, by reducing the duty cycle, the ion bombardment could be cut down leading to less dense films. Consequently, the mass deposition rate might remain stable while the thickness growth rate should increase. One more hint to explain this slight difference in growth rate could be found in XPS data related to carbon composition in the bulk. Indeed, by increasing the duty cycle from 25 to 100% the C content diminished from 3.4 to 2.5%.

### Influence of pulsed mode for 25% Ar–75% O_2_ gas mixture

Pulsing the plasma allows controlling the temperature of the substrate but is detrimental for the synthesis of highly efficient photocatalytic films. One final step toward process optimization has consisted in tuning the composition of the gas discharge. It has been shown, by other deposition technologies, such as RF magnetron sputtering^35^ or PE-ALD^12^*,* that there is an optimal composition of the Ar–O_2_ mixture to synthesize anatase crystals. Assuming that providing more O_2_ in the gas discharge will provide more active oxygen species, thus fostering TTiP oxidation in the vapour phase and/or on the substrate surface reactions, other film deposition were carried out. A study of the Ar–O_2_ mixture composition (from 0 to 100% O_2_) was carried out (not shown) and gave us an optimum condition for the degree of crystallization and photocatalysis at 75% O_2_ in the discharge. The results obtained in the 25% Ar–75% O_2_ mixture are now discussed.

The following deposition were led for 1 h under 500 W in a 25% Ar–75% O_2_ mixture with a set frequency of 10 kHz and a variable duty cycle.

Film thicknesses measured for a duty cycle of 25, 50 and 100% (765, 662 and 531 nm respectively) revealed that the growth rates in continuous and pulsed mode were a bit lower (≈ 9 nm/min) than these obtained with 75% Ar in the discharge (≈ 11 nm/min). The deposition rate is slightly higher when pulsing the plasma, similarly to what was previously observed with the 75% Ar–25% O_2_ mixture.

**Figure 6:**
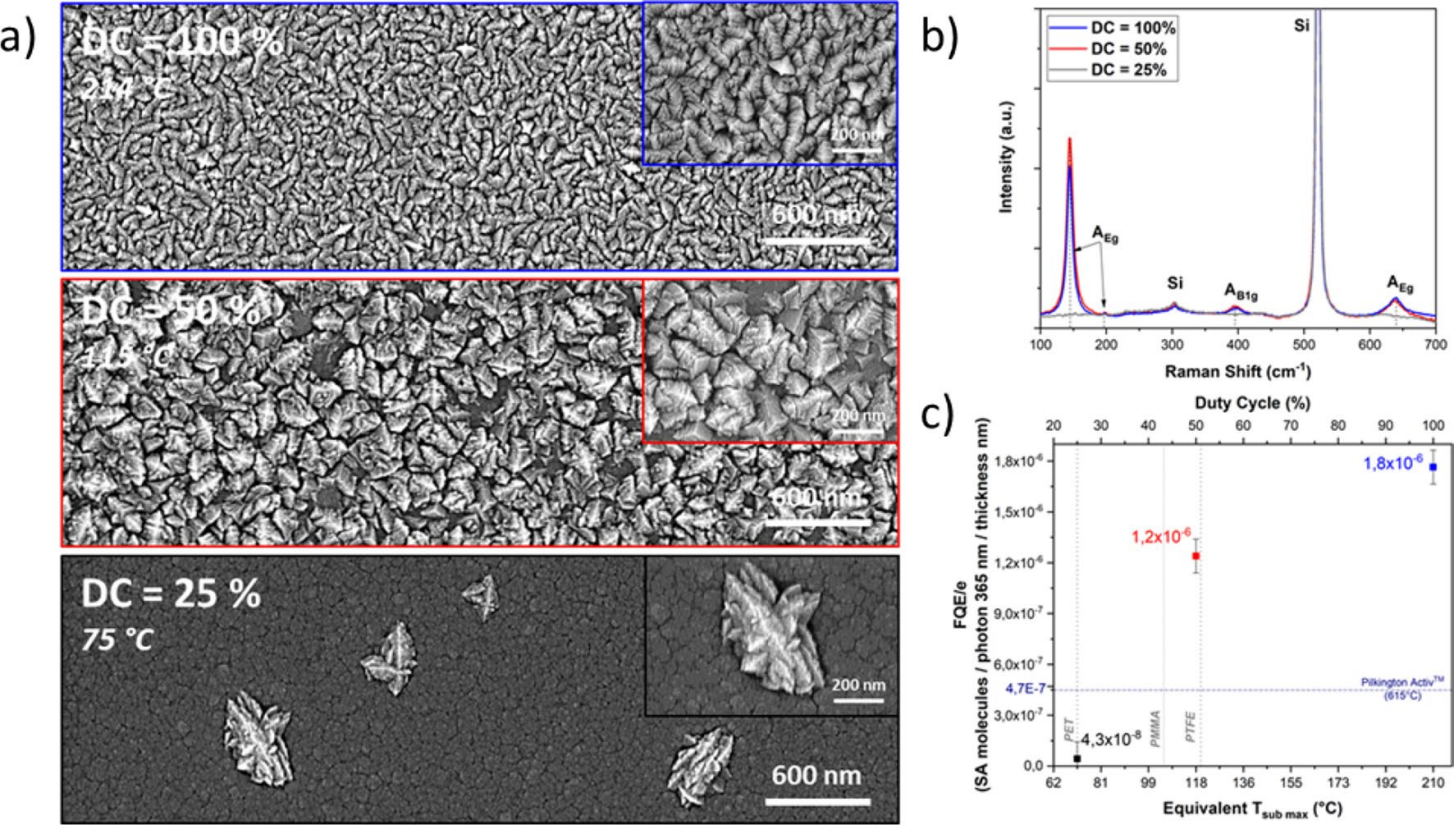
SEM topviews with associated T_sub max_ (**a**), Raman scattering spectra (**b**) and Photocatalytic activity normalized to the thickness (**c**) of TiO_2_ thin films deposited at different duty cycle (Power 500 W (T_on_), 1 h deposition, f = 10 kHz, 25% Ar–75% O_2_).

Great influences of the gas discharge composition on the growth deposition mechanism were here pointed out by SEM top views observations and Raman analysis Fig. 6. The first observation concerns the deposition with DC = 25% (T_sub max_ = 75 °C). Despite no Raman peaks related to anatase could be spotted out Fig. 6, few growing crystallite hillocks are emerging out of a smooth underlayer. The film obtained with DC = 50% (T_sub max_ = 115 °C) shows a top layer with high density of faceted flowers which is correlated with anatase characteristic peaks emergence on the Raman spectrum. A direct comparison between coatings deposited in continuous (T = 214 °C) and with DC = 50% revealed a similar Raman signature indicating an equivalent amount of anatase for each.

Figure 6 reports the results of the coatings normalized photocatalytic activities FQE/e as a function of the duty cycle. Since a linear dependency exists between the duty cycle and T_sub max_ both were displayed in the figure. In order to give better insight on the possible polymers that can be treated with the optimized pulsed-ECWR plasma process, glass transition of three polymers (PET: Polyethylene terephtalate, PTFE: Polytetrafluoroethylene, PMMA: Poly(methyl methacrylate) were also displayed. Interestingly, TiO_2_ coating synthesised at DC = 50% exhibited a high FQE/e of 1.2 × 10^–6^ nm^−1^ at a substrate temperature as low as 115 °C with a quality twice higher than the CVD reference Aktiv (FQE/e = 4.7 × 10^–7^ nm^−1^) obtained at 615 °C.

**Figure 7:**
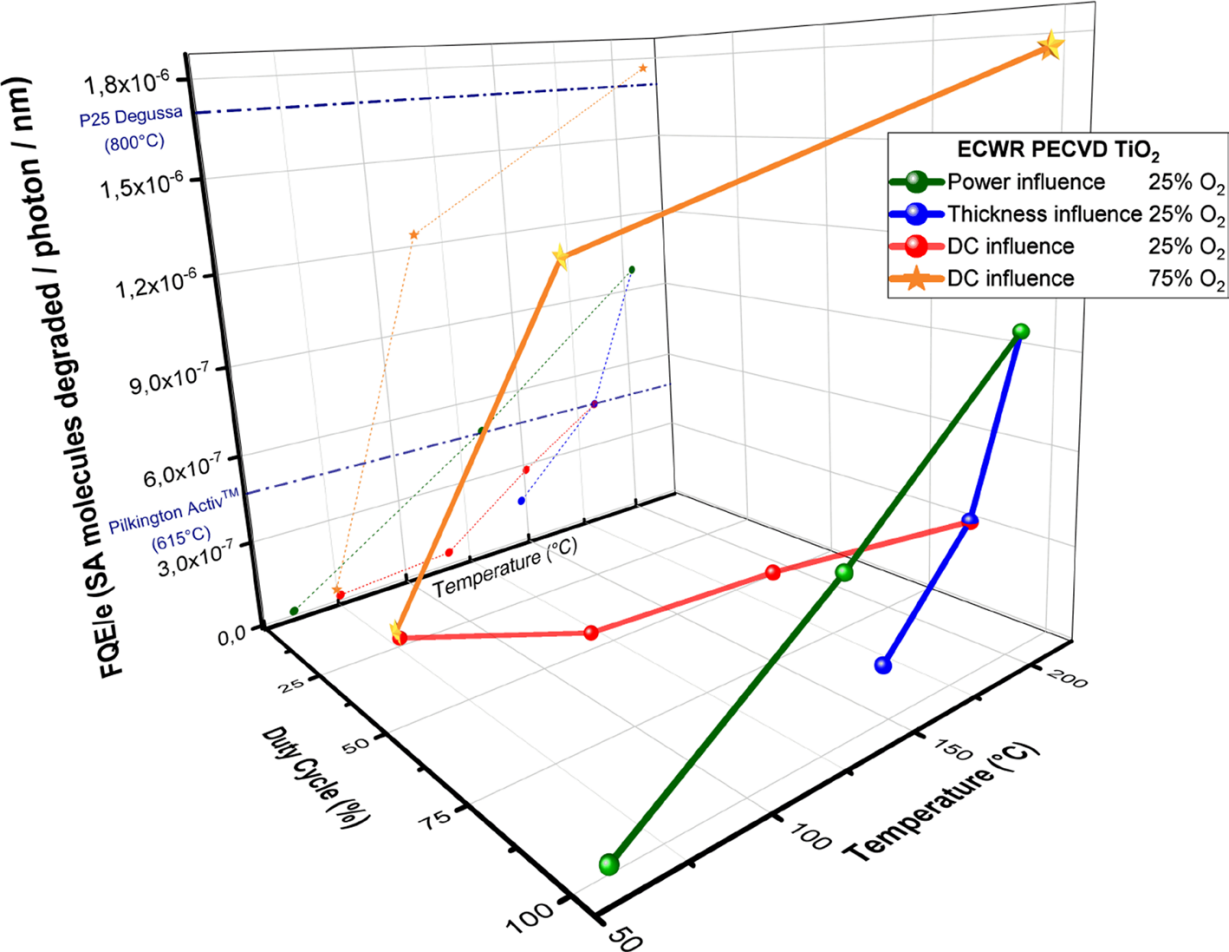
Summary of Normalized Photocatalytic Efficiency (SA molecules/photon 365 nm/coating thickness) for all samples obtained during the optimization study and values of references in the field^23^.

Therefore, by tuning the duty cycle, one can adapt the deposition temperature to meet the substrate requirements. In order to have a better overview of our samples specific efficiency in direct comparison with two commercial references in the field (P25 Degussa and Activ) all the FQE/e are regrouped in Fig. 7. They are displayed regarding the Duty Cycle and the final temperature of each samples.

The study on the power input in the plasma revealed that the higher the plasma power, the better the degree of crystallinity. Greater RF power induced higher electron density and ionic current density. However the increase of RF Power from 100 to 500 W induced a jump in T_sub max_ from 60 to 204 °C. Thicker films exhibited also an increase in the crystallinity and thus an increase of the photocatalytic property. It turned out that only 1 h deposition was required to get a highly cristallized and faceted coating covering the entire substrate reaching eventually a temperature of 183 °C. High FQE/e was obtained for that particular sample reaching a value twice higher that the one of Pilkington Aktiv.

However, in order to tune down the substrate temperature while maintaining a significant amount of anatase in the layer, pulsed-plasma with variable duty cycle were investigated. We noticed that growth happened in both T_ON_ and T_Off_ supposing the superposition of two different growth mechanisms involved in pulsed mode. Nonetheless, anatase phase could not be significantly synthesised for duty cycle below 75%.

Finally the increase of O_2_ up to 75% in the Ar–O_2_ mixture enabled the deposition of two very promising samples. First, for a continuous mode deposition with a final substrate temperature of 214 °C a FQE/e comparable with the one of P25 Degussa was obtained. Last, by tuning the duty cycle from 100 to 50% the T_sub max_ was lowered down to 115 °C making the deposition of highly crystalline layer possible on stiff polyethylene substrate (Fig. 1).

One can note that the substrate temperature in pulsed mode is low compared to other PECVD processes that lead to crystalline anatase coatings. Pulsing the plasma at DC = 50% roughly decreases by half the energy coupled to the plasma, thus limiting the heat transfer to the substrate. Nevertheless, despite this low temperature of the substrate, anatase TiO_2_ crystallites are formed. It is much likely that the specific plasma properties offered by the ECWR source play a major role in the crystallisation process using pulsed plasma.

Indeed, the presence of a grounded extraction grid at the outlet of the ECWR plasma source induces the diffusion of a “quasi neutral plasma beam” (composed of neutrals, electrons and ions) that flows out into the deposition chamber. Hence, ions and electrons are extracted from the plasma source and impinge the substrate. Ion energy distribution function (IEDF) measurement with a retarding field energy analyser (RFEA) evidenced positive ions with kinetic energy up to 30 eV in the deposition chamber. These energetic charged species when impinging the substrate may induce increased mobility of the adsorbed species on the growing surface. Therefore, TTIP fragments (TiO_x_C_y_H_z_) reaching the surface can more easily diffuse on the on-growing film surface facilitating the removal of organic ligands.

In addition, ECWR plasma sources are known to provide a high dissociation degree for diatomic gases, (i.e. up to 80%^36^) Experimentally, the production of atomic O is assessed by OES observation (emission of atomic O* at 777 and 845 nm). It is believed that atomic O produced during plasma-on times plays a significant role on the crystallisation mechanisms. Indeed, atomic oxygen recombination (O + O → O_2_) on a surface is an exothermic process known to provide a large quantity of energy. This recombination process may thus locally release a significant amount of energy on the coating surface, thus enabling crystallisation processes on the growing TiO_2_ thin film topmost surface.

The understanding of deposition and crystallisation mechanisms of TiO_2_ coatings in ECWR plasma and the pulsed plasma influence is a major point of interest, from a scientific and technical point of view. A detailed study is currently ongoing and would be published later.

## Conclusions

In this work, we presented an ECWR PECVD process able to readily deposit well crystallized anatase TiO_2_ without the need of any post-treatment. By carefully optimising the deposition parameters, anatase containing TiO_2_ layers with a thickness growth rate of 10 nm/min and a PCA twice higher than the Aktiv reference were obtained at substrate temperature below 115 °C.

In addition, it looks like films grown under pulsed mode conditions revealed a slightly higher growthrate. Pulsed mode showed also an interesting behaviour while it came to plasma gas composition. Indeed in case of 75% Ar–25% O_2_ mixture, one can notice that the substrate temperature decreased from 183 °C (CW mode) to 117 °C (DC = 50%) and the anatase phase is not created. However, by carefully tuning the amount of O_2_ in the discharge and the Duty Cycle we managed to reduce the temperature from 214 °C (CW mode) to 115 °C (DC = 50%) while keeping a huge amount of anatase. This study is a first step toward a better understanding of the growth mechanisms of TiO_2_ layers under pulsed plasma conditions.

## Methods

### Plasma enhanced chemical vapor deposition process

The experimental set-up used was equipped with a low-pressure radiofrequency (13.56 MHz) **E**lectron **C**yclotron **W**ave **R**esonance **P**lasma **E**nhanced **C**hemical **V**apour (ECWR-PECVD) source (COPRA DN 200 CF, CCR Technology)^13^. This specific plasma source consists in a single turn RF driven electrode and a low transverse static magnetic field ($\left|\overrightarrow{B}\right|\hspace{0.17em}$≈ tens of Gauss) allowing the creation of high-density plasma even at low pressure^36^. A grounded tungsten grid at the exit of the source allows confining the plasma. A quasi-neutral plasma beam (composed of electron, ions and neutral species) diffuses out of the source, through the extraction grid, into the deposition chamber and interacts with the titanium precursor before reaching the substrate surface. Titanium (IV) tetraisopropoxide (TTiP, Ti[OCH(CH_3_)_2_]_4_, 97 wt%, Sigma-Aldrich) was injected into the reactor chamber by a bubbler system at room temperature fed with Ar as a carrier gas. The pressure in the bubbler was maintained between 4.5 and 5.1 mbar. The gas mixture flow was composed of 7 sccm of Ar and 0.16 sccm of TTIP introduced into the deposition chamber through a dispersal ring between the ECWR plasma source and the stainless steel substrate holder. To reach this low TTiP flow the plasma source was additionally fed with a mixture of Ar and O_2_ with a flowrate of 50 sccm. The total pressure in the chamber was set at 5.10^–3^ mbar, thanks to a throttle valve installed between the deposition chamber and the turbomolecular pump. In this work, TiO_2_ films were deposited on intrinsic silicon substrate (100), thickness = 280 µm, single side polished). The substrate temperature was measured thanks to a K-thermocouple stuck at the back of each substrate. The ECWR plasma source can work both in continuous and pulsed modes. For this study, both modes were used and a frequency of f = 10 kHz was applied for the pulsed mode.

### Film characterization

The surface morphology was investigated by Scanning Electron Microscopy performed on a Hitachi SU-70 FE-SEM. The crystalline structure of the films was analysed using a Renishaw inVia micro-Raman spectrometer. The power used was around 2.6 mW on a spot of 1 µm^2^ with a 532 nm wavelength green laser. The thickness was determined either by SEM cross section and by profilometry by applying a 2 mg force to the stylus for each measurement. The surface and the bulk chemical composition were assessed by X-Ray Photoelectron Spectroscopy on a Kratos Axis Ulltra DLD. A monochromatic Al Kα X-ray source (hν = 1486.6 eV, 20 eV pass energy) for high-resolution spectra was used. The energy calibration was performed on adventitious carbon at 285.0 eV. An Ar^+^ beam with Energy of 4 keV and a current of 1.6 µA sputtered the sample for 600 s in order to obtain the bulk composition.

### Functional properties

The photocatalytic activity of the as-deposited TiO_2_ films was evaluated by measuring the stearic acid (SA) decomposition under UV exposure (Herolab UV-8 SL, 365 nm, 16 W). In order to deposit stearic acid on the entire surface of the sample, this organic compound was diluted in methanol (0.05 mol L^−1^) and then deposited by spin coating. A droplet of 6 µL was deposited on the TiO_2_ coatings (surface 1 cm^2^) with a spin-coating equipment during a time of 30 s at 6000 rpm. The samples were then stored in dark into a black box during one night before being exposed to the UV light source. The UV lamp was positioned at 10 cm above the samples. In order to precisely know the irradiance during exposition of each sample, a measurement was carried out at each corresponding sample position (φ_i_ mW/cm^2^) by a radiometer. The decomposition of the stearic acid was then quantified using a FTIR spectroscopy analyser (Bruker VERTEX 70 in transmission mode) by measuring the integrated area of absorption bands in the range 2700–3000 cm^−1^ (C-H stretching modes of the stearic acid). The photodegradation efficiency is determined through the monitoring of this absorption band area as a function of UV illumination duration. The linear regression of the area band gives a degradation rate (cm^−1^ h^−1^) which illustrates the photocatalytic efficiency. Besides, the FTIR absorption in the range 2700–3000 cm^−1^ is linked to the quantity of SA by a conversion factor δ, which gives an equivalency between a number of molecules of SA per cm^2^ and an integrated FTIR area value of 1 cm^−1^. A conversion factor value of δ = 3.17 × 10^15^ molecules of SA.cm^−2^ per cm^−1^ of FTIR absorption^23^ is used in this article. This allows us to calculate a Formal Quantum Efficiency defined as the number of molecules of SA degraded per photon.

1 $$FQE= {Slope}_{FTIR}\times \delta \times \frac{{E}_{photon}(365 nm)}{{\varphi }_{i}}$$

In order to benchmark the photocatalytic properties of the as-grown TiO_2_ samples, their estimated FQE is compared to those of two reference samples extracted from the literature: a P25 Degussa coating (wet chemistry, 90 nm, T_substrate_ = 80 °C) and Pilkington Activ (Atmospheric Pressure Chemical Vapor Deposition, 15 nm, T_substrate_ = 615 °C) from the work of Mills et al*.*^23^.

The samples obtained in the present study are relatively thick (400–1300 nm). One can hypothesize here that the excellent FQE measured from our as-grown TiO_2_ layers could partly be explained by their quite important thickness. On the other hand, samples considered as references in the field of thin films photocatalyst are 15–90 nm^23^. Thus, a FQE normalized to the film thickness (i.e. FQE/e) can be calculated which could be a simple way to compare the normalized photocatalytic efficiency (SA molecules/photon 365 nm/coating thickness nm) of the films grown independently from thickness effect. Even though we know that Stearic Acid does not penetrate the TiO_2_ thin film for more than few tens of nanometer and UV might totally be absorbed after 200 nm in TiO_2_, we consider that FQE/e is a good factor to compare our ECWR plasma deposited films with references obtained with various methods.
